# Volatiles of Grape Inoculated with Microorganisms: Modulation of Grapevine Moth Oviposition and Field Attraction

**DOI:** 10.1007/s00248-018-1164-6

**Published:** 2018-03-10

**Authors:** Marco Tasin, Sebastian Larsson Herrera, Alan L. Knight, Wilson Barros-Parada, Eduardo Fuentes Contreras, Ilaria Pertot

**Affiliations:** 1 Integrated Plant Protection Unit, Department of Plant Protection Biology, Swedish University of Agricultural Science, 23053 Alnarp, Sweden; 2 Department of Sustainable Agro-Ecosystems and Bioresources, Research and Innovation Centre, Fondazione Edmund Mach (FEM), San Michele all’Adige, Italy; 3 USDA, Agricultural Research Service, 5230 Konnowac Pass Rd, Wapato, WA 98951 USA; 4 Millennium Nucleus Center in Molecular Ecology and Evolutionary Applications in the Agroecosystems (CEM), Facultad de Ciencias Agrarias, Universidad de Talca, Casilla 747, Talca, Chile; 5 Escuela de Agronomìa, Facultad de Ciencias Agrarias y de los Alimentos, Pontificia Universidad Católica de Valparaíso, Casilla 4-D, Quillota, Chile; 6 Center Agriculture Food Environment (C3A), University of Trento, San Michele all’Adige, Italy

**Keywords:** *Lobesia botrana*, Acetic acid, 2-phenylethanol, Dual sex attractant, Pest monitoring

## Abstract

Semiochemicals released by plant-microbe associations are used by herbivorous insects to access and evaluate food resources and oviposition sites. Adult insects may utilize microbial-derived nutrients to prolong their lifespan, promote egg development, and offer a high nutritional substrate to their offspring. Here, we examined the behavioral role of semiochemicals from grape-microbe interactions on oviposition and field attraction of the grapevine moth *Lobesia botrana* (Denis & Schiffermüller). The volatile constituents released by grape inoculated with yeasts (*Hanseniaspora uvarum* (Niehaus), *Metschnikowia pulcherrima* (Pitt.) M.W. Miller, *Pichia anomala*, *Saccharomyces cerevisiae* Meyen ex E.C. Hansen, and *Zygosaccharomyces rouxii* (Boutroux) Yarrow), sour rot bacteria (*Acetobacter aceti* (Pasteur) Beijerinck and *Gluconobacter oxydans* (Henneberg) De Ley), and a fungal pathogen (*Botrytis cinerea* Pers.) all endemic of the vineyard were sampled by solid-phase microextraction and analyzed by gas-chromatography coupled with mass spectrometry. Ethanol, 3-methyl-1-butanol, and ethyl acetate were the most common volatiles released from all microbe-inoculated grapes. In addition, acetic acid was released at a substantial amount following bacteria inoculation and in a three-way inoculation with yeasts and the fungus. 2-phenylethanol, a compound reported to attract tortricid moths when used in combination with acetic acid, was found at a relatively low level in all microbial combinations as well as in the control grape. While grapes inoculated with a consortium of yeasts stimulated oviposition in comparison with uninoculated berries, the phytopathogenic fungus deterred egg-laying. Nonetheless, the highest preference to lay eggs was measured when the yeasts were co-inoculated with the fungus. The lowest preference was obtained when grapes were inoculated with sour rot bacteria and their binary co-inoculation with yeasts and the fungus. Interestingly, oviposition on berries simultaneously inoculated with all the three microbial groups was unaffected. Lures loaded with either acetic acid or 2-phenylethanol were not attractive when placed in traps as single component in vineyards, but a binary blend attracted both sexes of grapevine moth in significant numbers. Further addition of the three most common volatiles released by infected berries (ethanol, 3-methyl-1-butanol, and ethyl acetate) did not significantly increase moth catch with this binary blend. The ecological implications of the grape-microorganism and grapevine moth interaction as well as the possibility to develop a pest monitoring system based on microbial volatiles are discussed.

**Electronic supplementary material:**

The online version of this article (10.1007/s00248-018-1164-6) contains supplementary material, which is available to authorized users.

## Introduction

Olfactory cues emitted by plant-microbe associations are utilized by a number of insects to locate food resources [1]. In comparison with other sensory cues such as visual or tactile stimuli, olfactory cues can be sensed over large distances and are likely to play an ecological role within the triple plant-microbe-herbivore interaction. In herbivorous insects with plant-feeding larval stages and a non-feeding adult stage, the quality of the food consumed during pre-imaginal stages settles the reproductive output of the adults. Microorganisms can affect such performance by changing the nutritional value of the plant on which they grow. This process is accompanied by a simultaneous shift in the volatile profile of the plant, which will carry not only plant compounds but also de-novo synthetized microbial components.

Microbial compounds can attract insects to infected plant with an increased content of vitamins, protein, and other nutrients, which adult insects utilize to prolong their lifespan, to increase their resistance against parasitoids, to promote egg development, and to offer a high nutritional substrate to the offspring [2, 3]. The ecological function of microbial food-signaling volatiles has been studied, but the utility of these compounds as attractant to monitor or mass trap insect pests has been explored for only a few species [1, 4–8].

Several studies have evaluated the use of microbial volatiles from fermenting baits to survey moths, and noctuids have consistently been the most common species group collected [9–11]. However, more recent studies have focused on the attraction of various tortricids to microbial volatiles, including key horticultural pests, such as the codling moth *Cydia pomonella* (L.) and the summer fruit tortrix *Adoxophyes orana* (Fischer von Röslerstamm) [12, 13]. Less information is available for a number of pests of other economically important crops such as grapevine.

In this study, we examined the effect of microbial volatiles on the grapevine moth *Lobesia botrana* (Denis & Schiffenmüller). *Lobesia botrana* is a polyphagous herbivore associated with grapevine *Vitis vinifera* (L.). While oviposition, larval and wind tunnel attraction of grapevine moth to host plant volatiles, and their physiological response were established and confirmed through several studies [14–17], the response to microbial volatile metabolites has been the object of more recent investigations. In vineyards, due to a diverse range of microorganisms that may infect the grapes, *L. botrana* larvae and adults are attracted to berries with a highly variable nutritional value. Both oviposition and larval fitness were substantially affected by these microorganisms [18, 19], with larvae being involved in spreading a fungal pathogen of grape [20].

A large variation among the volatile composition of single microorganism headspace and their effect on moth oviposition was measured. While yeasts (*Hanseniaspora uvarum* (Niehaus), *Metschnikowia pulcherrima* (Pitt.) M.W. Miller, *Pichia anomala*, *Saccharomyces cerevisiae* Meyen ex E.C. Hansen, and *Zygosaccharomyces rouxii* (Boutroux) Yarrow) were found to stimulate egg deposition, the phytopathogenic fungus *Botrytis cinerea* Pers. and the bacteria associated with grape rot (*Acetobacter aceti* (Pasteur) Beijerinck and *Gluconobacter oxydans* (Henneberg) De Ley) triggered the opposite effect [18]. In vineyards, microorganisms such as fungi, yeasts, and bacteria co-occur often at the grape surface [21]. However, the possible effect of combinations of these microorganisms on the behavior of the herbivore has not previously been considered. Similarly, the volatile profile of berries in the field exposed to a diverse microbial inoculation has not previously been characterized.

Here, we identify the volatiles released by grape berries infected with different combinations of the abovementioned microorganisms endemic of the vineyard using solid-phase microextraction (SPME) coupled to gas-chromatography and mass spectrometry (GC-MS). Second, we compared the level of oviposition on infested berries in a laboratory choice test against uninoculated and sterilized berries. Third, we evaluated the potential attractiveness of various volatile blends to *L. botrana* in a field setting.

## Material and Method

### Insects and Microorganisms

*Lobesia botrana* was originally collected in Italy and maintained in the laboratory on a semi-artificial diet at 25 °C, 70% relative humidity, and under a 17:1:6 h light/dusk/dark photoperiod. Field-collected larvae were grown to adulthood and the following offspring have been added to this colony each year to minimize an inbreeding effect [18]. The microorganisms used in this study were isolated from untreated vineyards in Trento (Italy) as described in an earlier study [18]. We tested a consortium of five yeasts (*S. cerevisiae*, *Z. rouxii*, *M. pulcherrima*, *H.uvarum*, and *P. anomala*) commonly present on ripe berries; two species of bacteria (*G. oxydans* and *A. aceti*) commonly isolated from berries showing sour rot symptoms; and *B. cinerea*, the phytopathogenic fungus causing gray rot. Ripe grapes (*V. vinifera* cv. Pinot gris) were randomly collected from an untreated vineyard in Trento (Italy). Five replicates of ten berries each were washed by dipping for 10 min in 50 ml of sterile water with 0.01% Tween 80 (polyoxyethylene sorbitan monooleate, Acros Organics, Geel, Belgium). The suspensions were then serially diluted and plated on potato dextrose agar (PDA; Oxoid, Milan, Italy). Morphologically different colonies were selected and identified at specie level based on morphological, biochemical, physiological, and molecular approaches [22, 23]. One isolate for each of the yeast species found (*H. uvarum*, *M. pulcherrima*, *P. anomala*, *S. cerevisiae*, and *Z. rouxii*) was selected and maintained on PDA at 5 °C until use. Isolates of two species of acetic acid bacteria (*G. oxydans* and *A. aceti*) were selected and maintained on LPGA (Oxoid). *Botrytis cinerea* was isolated from grapes (*V. vinifera* cv. Cabernet Sauvignon) with gray mold in the same vineyard and maintained on PDA at 5 °C until use.

### Grape Inoculation

The inoculation of berries was carried out at FEM (Italy) following a published protocol [18]. Briefly, 100 intact ripe berries cv. Waltham were surface-sterilized with sodium hypochlorite (1%; Sigma-Aldrich, Milan, Italy) for 5 min and thereafter washed twice in sterile water. Five evenly distributed wounds (~ 2.0 mm) were inflicted on the longitude of each berry with a sterile scalpel. The abovementioned isolates were grown on the respective media in Petri dishes for 5 to 7 days at 25 °C. Suspensions of cells were collected with 5 mL of sterile distilled water, and cell concentration was adjusted to 1 × 10^6^/mL for yeasts and 1 × 10^7^/mL for bacteria by dilution, after counting the yeast cells under the microscope in a Thoma cell and by estimating the bacterial cells by reading the optical density (OD_600_) with the spectrophotometer. The adjusted suspensions were then mixed in equal proportion to obtain two suspensions (consortia of the yeasts and the bacteria) Berries were then inoculated by placing a drop (5 μL) of each microbial suspension. The following combination of suspensions were carried out: consortium of yeasts, consortium of bacteria, *B. cinerea*, consortium of yeasts + consortium of bacteria, consortium of yeasts + *B. cinerea*, consortium of bacteria + *B. cinerea*, consortium of yeasts + consortium of bacteria + *B. cinerea*. Berries wounded and treated with a drop of sterile distilled water served as untreated control. For *B. cinerea*, a small portion of mycelium was placed on the wounds. Inoculated and control berries were placed separately in sterile Petri dishes on wet filter paper (three berries per dish), covered by a pierced plastic cup, sealed with parafilm, and incubated for 16 h at 22 °C and 99% RH. At the end of the incubation, berries were used in the oviposition bioassay as odor stimulus. Plastic cups (61 mm base diameter × 88 mm top diameter × 130 mm high) served as oviposition devices and were assembled to avoid any physical contact of the insect with the berry. Cups and all materials used for experiments were glove-handled to avoid any contamination and disposed after each single use.

### Analysis of the Odor Profile

**Fig. 1:**
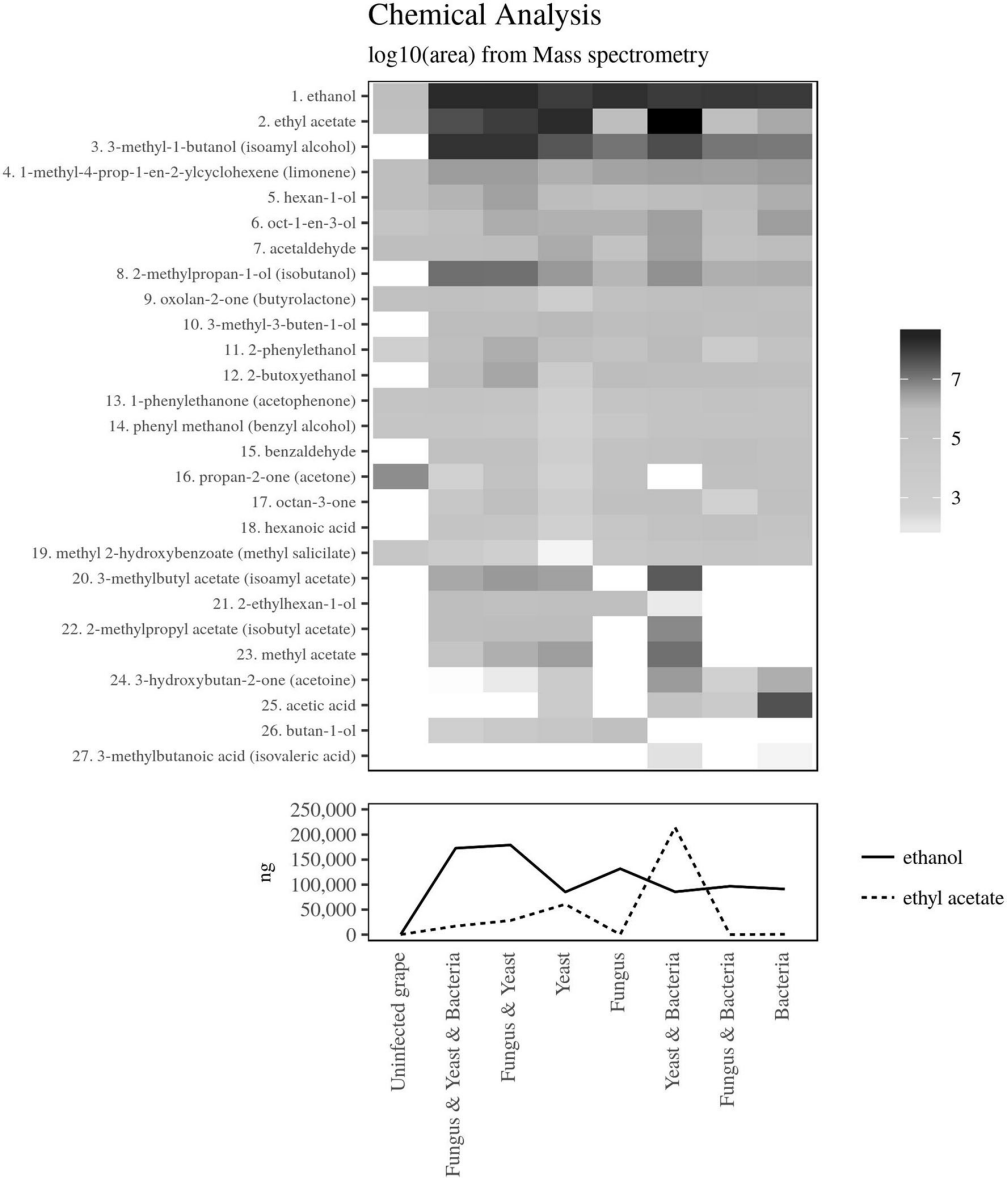
Heat map representing the chemical analysis of volatile compounds emitted by single or multiple microorganisms inoculated on grapes. Compounds were identified via SPME-GC-MS. The scale of the heat map represents a log 10 value of the compound abundance. The calibration of the SPME efficiency is shown in the graph at the bottom

Following the incubation time described above, volatiles emitted from uninoculated berries and from berries inoculated with *B. cinerea*, yeasts, acetic bacteria and their binary and ternary combinations were collected by solid-phase microextraction (SPME). Six berries with visible successful inoculations were randomly selected from each batch and placed into a 100-ml glass jar, with an opening closed by a single layer of parafilm^©^ for each collection assay. Following an equilibration time of 30 min, volatiles in the jar were adsorbed by a SPME fiber previously conditioned at 250 °C for 5 min in a gas-chromatograph injection port (triphasic fiber SPME, 2 cm length, film thickness 50/30 μm, coating divinylbenzene/carboxen/polydimethylsiloxane; Supelco, USA). After a collection time of 60 min, volatiles collected on the fiber were desorbed and injected in a gas-chromatograph coupled to a mass spectrometer (GC-MS, Clarus 500, Perkin Elmer, Waltham, USA) equipped with an Innowax column (30 m × 0.32 mm × 0.5 μm, Agilent, Palo Alto, USA). The SPME fiber was desorbed in splitless mode for 5 min in the GC injector port at 250 °C. The GC oven was programmed at 40 °C for 3 min, raised from 40 to 180 at 4 °C min^−1^, 180 °C for 4 min, raised from 180 to 220 at 10 °C min^−1^, and held at 220 °C for 10 min. Helium was used as carrier gas with a constant flow of 1.5 mL min^−1^. The temperature of the transfer line was set at 250 °C. The mass spectrometer operated in electron ionization mode (EI, internal ionization source; 70 eV) with a scan range between m/z 30 and 300. A calibration of the SPME collection efficiency was carried out for the compounds ethanol and ethyl acetate by using synthetic standards (Anfora et al. 2005). Results were used to calculate the amount release by each treatment (Fig. 1). The GC-MS database were analyzed using the Agilent MS software version 4.1 (Agilent, Santa Clara, USA). Compounds were identified by comparing their spectra with those of Wiley library as well as by comparing their Kovats retention indices with those published in literature. Kovats index of compounds was based on retention times of a blend of reference hydrocarbons. All identified compounds were injected as synthetics to calculate their Kovats index.

### Oviposition Bioassay

**Fig. 2:**
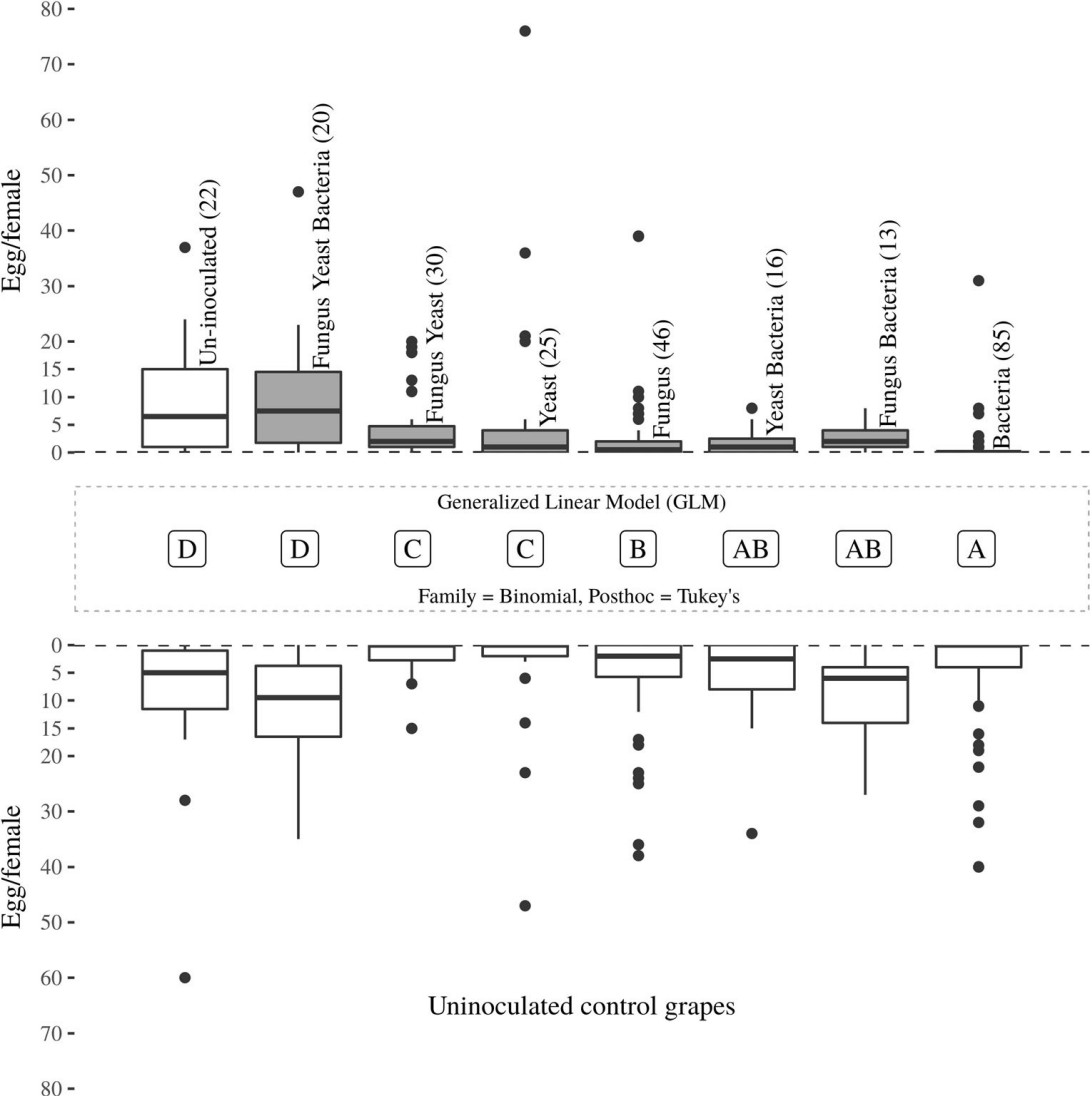
Boxplot representing the number of eggs laid by *L. botrana* females a laboratory dual-choice experiment with uninoculated or microorganism inoculated grapes. Choice experiments were done in net-cages. Non-respondent insects were included in the statistical model. The boxplot includes the median line (tick line inside the box), the interquartile range (lower and upper box limits), the variability outside the interquartile range (whisker), and the outliers (points). Letter in the middle box indicates significant difference based on the number of eggs laid at each side of the bioassay and their ratio

Oviposition preferences of *L. botrana* females were conducted at FEM (Italy) with each of the seven types of inoculated versus uninoculated *V. vinifera* grapes in a series of choice assays conducted in cylindrical net-cages (25 cm diameter, 50 cm long, 1.5 mm mesh). Following emergence, a male and a female were confined for 24 h into a plastic container to mate. Only 1–2-day-old females that laid eggs were used in bioassays. Oviposition assays were conducted under the same climatic conditions of the rearing. A 2-day-old mated female was released into the center of each cage. Mated females were allowed to choose between two oviposition substrates confined into a cage at a distance of 30 cm. After 72 h, moths were removed and laid eggs counted. The replication of each oviposition choice experiment is presented in Fig. 2.

### Field-Trapping Experiment

Through an exploratory experiment carried out in a vineyard in Verona (Italy) with a moderate population of grapevine moth, we found that a lure releasing ethyl acetate, 3-methyl-1-butanol, ethanol, 2-phenylethanol, and acetic acid attracted more moths than a blank trap. Although this result was not supported by a statistical significance, we decided to further challenge the potential of these compounds in a larger field-trapping test with a higher population of the target pest. Our attention focused on the major common volatiles (ethyl acetate, 3-methyl-1-butanol, ethanol, and acetic acid) and on 2-phenylethanol, a microbial and plant volatile reported in literature as moth attractant [24–27]. A field test in the Maule Region (Chile) was therefore conducted during February and March 2017 in a “Cabernet Sauvignon” vineyard situated near Molina (35° 04′ 14.29″ S, 71° 15′ 17.92″ W). Vines were planted at a density of 1110 plants ha^−1^ with a “tendone” trained 2.3 m tall canopy. The vineyard was managed with mating disruption for *L. botrana* using Isonet L (Shinetzu, Tokyo, Japan) at 500 dispensers ha^−1^. No insecticides were sprayed during the experiment. Orange delta traps (Süsbin, Mendoza, Argentine) with hot melt pressure adhesive liners (Alphascent, West Linn, OR, USA) were used to monitor *L. botrana*. Volatile compounds were loaded in 1.5 mL microcentrifuge plastic tubes (Sorenson BioSciences, Salt Lake City, UT, USA), termed from now on “lures,” with a 1-mm perforation hole in the lid, which contained also a dental cotton wick to adsorb the solution. Blends of volatile compounds (Fig. 4) were kept cold on ice during lure loading to prevent evaporation. Volatiles were loaded as single compound or as a blend within a single lure, except for acetic acid, which was loaded in a different lure to prevent esterification of the alcohols present in the blends. Due to the particularly high volatility of the compounds, we increased the load of the lure in comparison to the exploratory trial. In accordance with data from literature [25, 28, 29], we chose a 500-mg load for acetic acid and a 7.5–30-mg load for the other compound (Fig. 4). After loading the cotton wick with the compound(s), 30 μl of mineral oil (Sigma-Aldrich, Saint Louis, MO, USA) was added on top of the volatile(s) and the cotton wick to slow down the evaporation rate (Knudsen et al. 2015). For acetic acid, 500 mg was loaded in the lure and no mineral oil was added. Lures were hung from the roof of the delta traps with a clip. Five trap replicates were randomly located in the canopy with a spacing distance of approximately 20 m on January 31, 2017. Lures were replaced weekly or every 2 weeks (acetic acid). Liners were inspected weekly, and trap location was rotated on each sample date until March 24, 2017.

### Statistics

Statistical analyses were carried out using R software [30] and results are presented in Table 1. Cook’s distance was used to investigate influential points as possible outliers in the chemical dataset. When a single data point deviated more than three times from the respective mean, it was counted as an outlier and removed from the dataset. The composition of the microbial odors is graphically presented as a heat map (Fig. 1 and Table S1 in the additional data). The quantification of ethyl acetate and ethanol in each microbial headspace was calculated using a linear model based on the correlation between area count from injections of synthetic amounts and SPME collections (R^2^ = 0.97 and 0.98 for ethanol and ethyl acetate, respectively).

**Table 1:** Output from the statistical analyses

Model		Distribution^a^	Dispersion	Estimate	SE	z	*P* value
Oviposition treatment vs control							
Uninoculated (control)		Negative binomial (0.569)	0.938	− 0.026	0.400	− 0.065	0.948
Fungus yeast bacteria		Negative binomial (0.861)	0.828	− 0.213	0.322	− 0.661	0.508
Fungus yeast		Negative binomial (0.429)	0.976	0.820	0.417	1.965	0.049
Yeast		Negative binomial (0.188)	0.978	0.604	0.656	0.922	0.357
Fungus		Negative binomial (0.302)	1.128	− 0.841	0.419	− 2.006	0.045
Yeast bacteria		Negative binomial (0.378)	0.720	− 0.995	0.517	− 1.927	0.054
Fungus bacteria		Negative binomial (1.051)	0.670	− 1.136	0.351	− 3.233	0.001
Bacteria		Negative binomial (0.133)	(1.368)	− 1.598	0.516	− 3.096	0.002
Oviposition pairwise comparison^b^		Binomial, cbind()	1				
Fungus yeast vs control				0.846	0.182	4.640	< 0.001
Yeast vs control				0.630	0.158	3.992	0.002
Fungus vs control				− 0.815	0.150	− 5.453	< 0.001
Yeast bacteria vs control				− 0.970	0.225	− 4.310	< 0.001
Fungus bacteria vs control				− 1.110	0.219	− 5.063	< 0.001
Bacteria vs control				− 1.572	0.167	− 9.391	< 0.001
Fungus yeast vs fungus yeast bacteria				1.033	0.179	5.773	< 0.001
Yeast vs fungus yeast bacteria				0.817	0.154	5.307	< 0.001
Fungus vs fungus yeast bacteria				− 0.628	0.146	− 4.319	< 0.001
Yeast bacteria vs fungus yeast bacteria				− 0.782	0.222	− 3.520	0.010
Fungus bacteria vs fungus yeast bacteria				− 0.923	0.217	− 4.263	< 0.001
Bacteria vs fungus yeast bacteria				− 1.385	0.164	− 8.456	< 0.001
Fungus vs fungus yeast				− 1.661	0.187	− 8.892	< 0.001
Yeast bacteria vs fungus yeast				− 1.815	0.251	− 7.223	< 0.001
Fungus bacteria vs fungus yeast				− 1.958	0.246	− 7.944	< 0.001
Bacteria vs fungus yeast			–	− 2.417	0.201	− 12.01	< 0.001
Fungus vs yeast				− 1.446	0.163	− 8.862	< 0.001
Yeast bacteria vs yeast				− 1.600	0.234	− 6.830	< 0.001
Fungus bacteria vs yeast				− 1.740	0.229	− 7.608	< 0.001
Bacteria vs yeast				− 2.202	0.180	− 12.25	< 0.001
Bacteria vs fungus				− 0.756	0.172	− 4.388	< 0.001
Multicomparison of field catches^b^							
Males	Blend 7 vs blend 2/3/4	Negative binomial (1.099)	0.661	2.944	0.968	3.043	0.019
	Blend 8 vs blend 2/3/4			2.708	0.972	2.785	0.040
Females	Blend 7 vs Blend 4	Negative binomial (0.809)	0.409	2.996	0.795	3.769	0.001
	Blend 8 vs blend 4			3.296	0.791	4.164	0.001
	Blend 7 vs blend 5			2.303	0.654	3.523	0.004
	Blend 8 vs blend 5			2.603	0.650	4.007	< 0.001

^a^Theta parameter for negative binomial distribution

^b^Only significant comparisons are shown

**Fig. 3:**
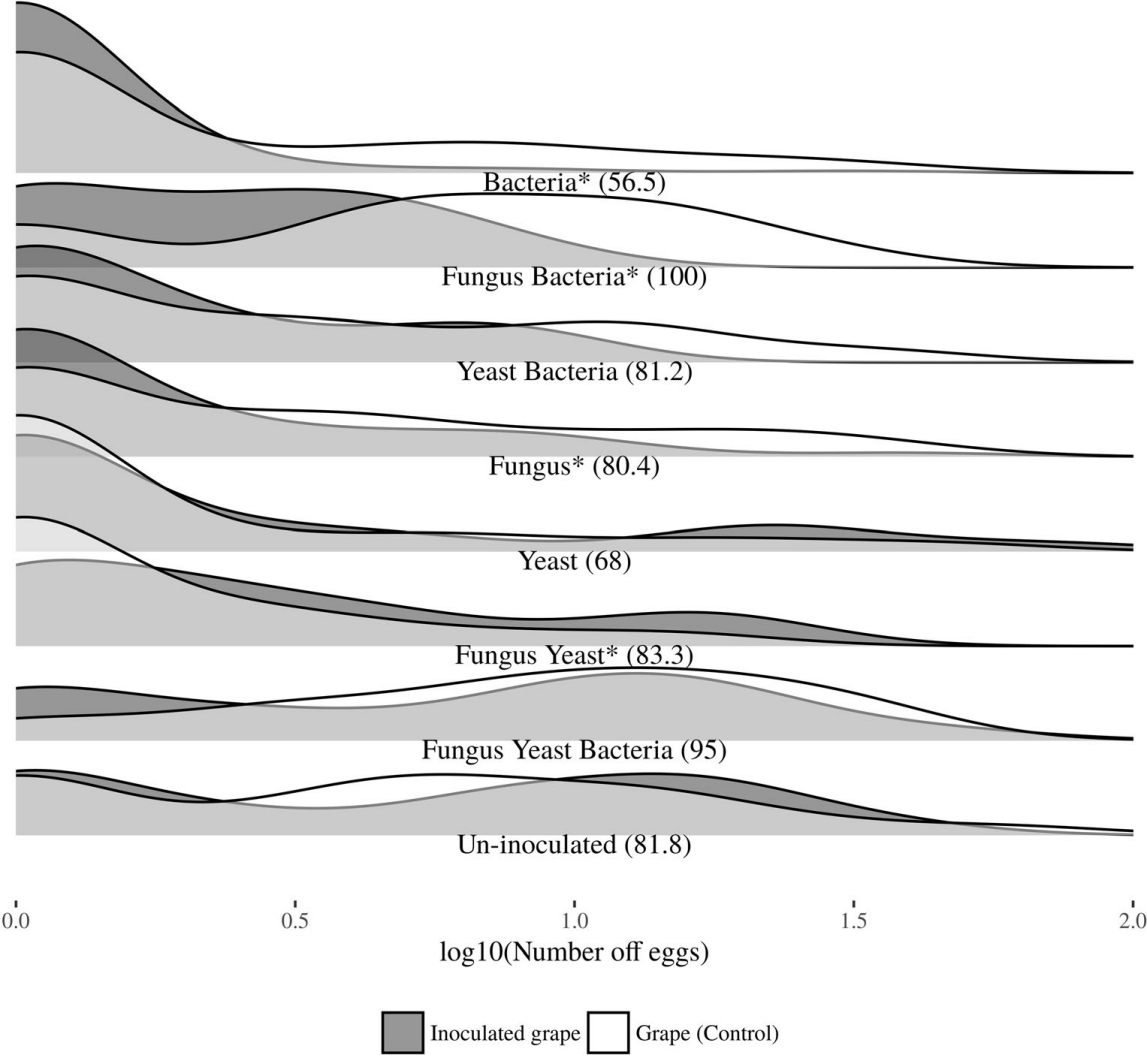
Density distribution of *L. botrana* egg in a laboratory dual-choice experiment with uninoculated or microorganism-inoculated grapes. The experiment was done in net-cages. Percentage of responding female is shown in parenthesis. The asterisk indicates a significant choice for one of the two treatments. The area delimited by each ridgeline is equal to 1

A density plot representing the number of laid eggs in the oviposition choice experiment was produced using the R package ggjoy 2.10 (Fig. 3). We used a density plot in order to avoid the stipulation of the data in bin width, which may lead to a skewed picture due to differences in replication. In the density plot, the overall area of each “ridgeline” is equal to 1. This gives the reader a direct understanding of the differences between egg distributions in each treatment. The whole dataset was used in this analysis, including non-responding insects.

In addition, oviposition choice data were also analyzed using a binomial generalized linear model with a cbind function. Through this analysis, it is possible to compare treatments with each other taking into consideration not only the amount of eggs laid at the inoculated side but also the ratio of eggs between the two choices. Data are presented as a box plot including outliers. Tukey’s post hoc test was used to discriminate between treatments (Fig. 2).

**Fig. 4:**
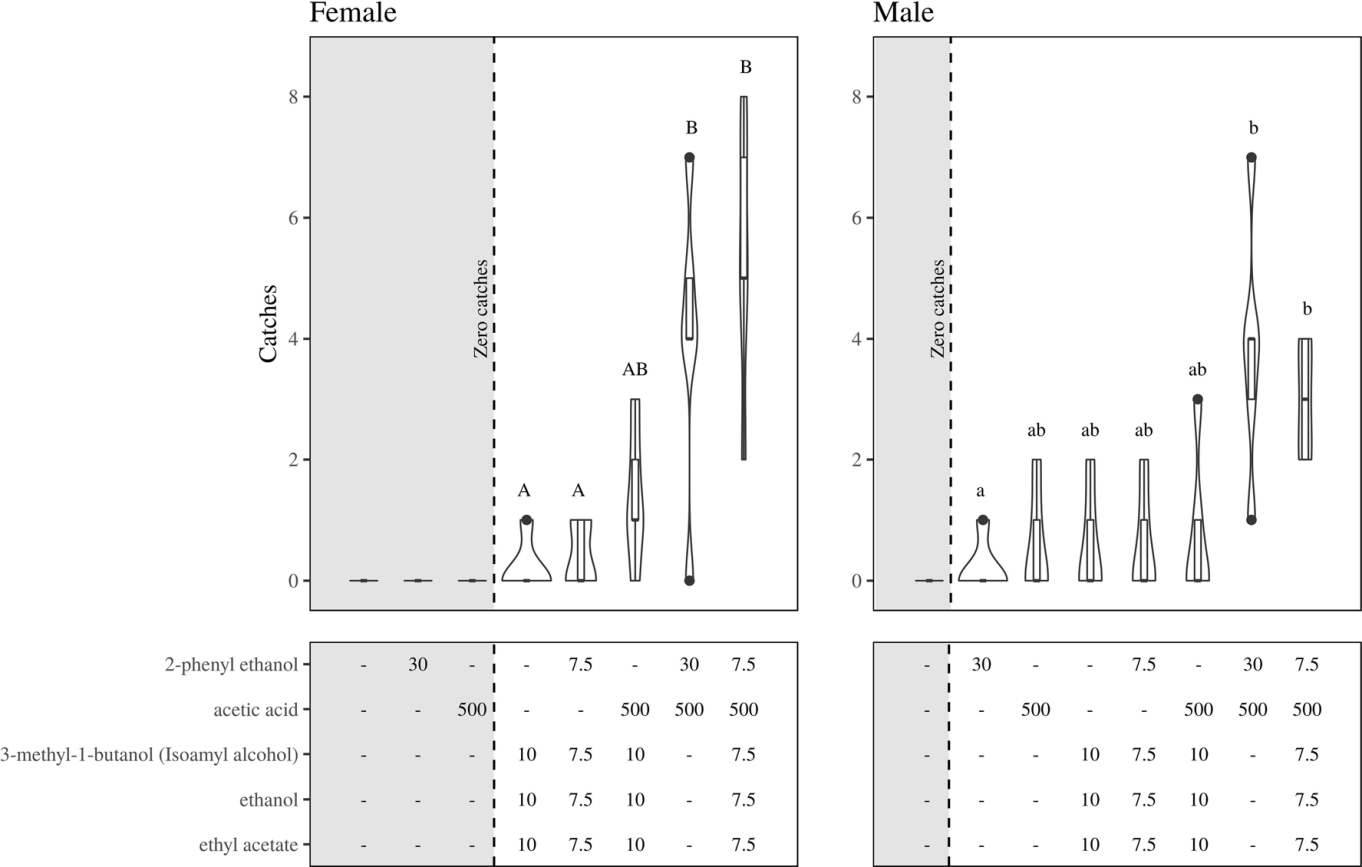
Boxplot with field catches of both sexes of *L. botrana* from a vineyard in Chile during 2017. A total of 57 females and 48 males were caught. The boxplot includes the median line, the 25 and 75% range (lower and upper box limits), and the outliers. The thickness of the bar mirrors the density of the catch at a given level. Treatments capped with the same letter do not differ significantly in the number of caught moths

The field dataset distributed according to a negative binomial family and was analyzed using the function glm.nb (library MASS). Because of our dataset did not fit into a zero-inflated model, treatments with no variance, i.e., with no catches, were excluded from the analyses. This allowed us to fit the data to a more accurate model. Treatments were separated by Tukey contrasts (Fig. 4).

## Results

### Analysis of Odor Profile

Volatiles released by grapes inoculated with microorganisms belong to the chemical classes of aldehydes, ketones, alcohols, acids, esters, lactones, terpenoids, and benzenoids (Fig. 1 and Table S1). The composition of the headspace showed a high variability among microorganisms. Ethanol and 3-methyl-1-butanol were identified as main components in all three categories of microorganisms. Ethanol, 3-methyl-1-butanol, and limonene were the major compounds identified in the headspace from grapes inoculated with the fungus. Ethyl acetate, along with ethanol and 3-methyl-1-butanol, was the major component released by the yeasts. Ethanol, acetic acid, and 3-methyl-1-butanol were the major volatiles from grape inoculated with the sour rot bacteria. Co-inoculating yeasts with the fungus resulted in a relative increase in 3-methyl-1-butanol, a reduction of ethyl acetate, and a total inhibition of acetic acid emission compared to the release of yeasts and the fungus alone. An increase in acetic acid emission was observed when the bacteria were added to the fungus, while the release of its precursor, ethanol, diminished. When bacteria were inoculated with yeasts, release of ethanol and acetic acid decreased while their corresponding ester ethyl acetate increased. The ternary combination showed a higher release of 3-methyl-1-butanol compared to each of the single microbial categories. Although released by the entire range of tested microbes, a higher proportion of 2-phenylethanol was measured in the headspace of yeasts and both binary and ternary combinations. While the bacteria and yeast co-inoculation released the highest absolute amount of ethyl acetate (214 ng per sample), the fungus and yeast co-inoculation followed by their combination with the bacteria emitted the highest quantity of ethanol (192 and 181 ng per sample, respectively). Uninfected wounded grapes release a number of plant volatiles such as hexan-1-ol, limonene, 1-octen-3-ol, benzyl alcohol, methyl salicylate, and 2-phenylethanol. Although to a much limited extent than infected grapes, compounds possibly associated with the wounding process such as acetone, acetaldehyde, ethanol, ethyl acetate, butyrolactone, and acetophenone were also released by the uninoculated grapes.

### Oviposition Bioassay

In Fig. 3, it is presented the egg density measured in each dual choice experiment. While grapes inoculated with the yeasts stimulated oviposition, the fungus deterred egg-laying. However, the highest choice to lay eggs was measured when the fungus was co-inoculated with the yeasts. This co-inoculation triggered a significantly higher number of eggs than the control grape. Repellence was observed when grapes were inoculated with sour rot bacteria or their combination with yeasts or the fungus. Grapes inoculated with all the three microbe categories were neither repellent nor attractive to grapevine moth females (see Table 1 for further details).

When comparing the different dual-choice experiments with each other through a GLM, it is possible to appreciate that the treatments including the bacteria and the one including the fungus alone triggered a significantly lower amount of eggs in comparison to the yeast and the yeast + fungus. These last two treatments stimulated a lower egg-laying than the three-way inoculum or the uninoculated grapes (Fig. 2). The higher number of eggs released at the side of the arena with the microbe-inoculated grape was measured for the ternary inoculation (9.7 eggs female^−1^), followed by the yeast consortium (7.8 eggs female^−1^). A lower number of eggs was laid when fungus plus yeasts were co-inoculated (4.8 eggs female^−1^) or at the stimuli with the sour rot bacteria and their combination with the yeasts or the fungus (2.1 and 2.8 eggs per female^−1^). Similarly, the fungus alone elicited a low oviposition (2.3 eggs per female).

### Field-Trapping Experiment

Blank traps did not catch any moth. While females were not attracted to traps baited with single components (acetic acid or 2-phenylethanol), a small number of males responded to those components (Fig. 4). When these two volatiles were presented in a unique blend, the response of both sexes increased, with a stronger effect in females. Although both sexes showed some attraction to a three-component blend of 3-methyl-1-butanol, ethanol, and ethyl acetate, no synergy occurred when acetic acid or/and 2-phenylethanol were added to this blend.

## Discussion

The chemical signals produced by the interactions of the grapes and microorganisms can be characterized by a set of major volatiles, including ethanol, ethyl acetate, acetic acid, and 3-methyl-1-butanol. However, the blends of these volatiles differ widely among the three groups of microorganism and are altered by the various binary and ternary combinations. Importantly, our laboratory oviposition assays demonstrate that these volatile bouquets have a strong behavioral effect impacting the utilization of the host plant resource by female *L. botrana*. Our preliminary field trial demonstrates that specific blends of microbial volatiles may be key cues used by both male and female moths to orient to the host plant.

Interestingly, a relatively minor but common volatile 2-phenylethanol when presented in combination with acetic acid was attractive to both sexes of moths. In addition, when presented with all of the major volatiles, this blend retained its attractiveness.

A change in host quality induced by a microbial infection may trigger a variation in volatile emission, which is sensed by herbivorous insect [31, 32]. An attempt to correlate food quality with attraction to food volatiles was done by Tasin et al. [18] for *L. botrana*. In particular, eggs laid on a yeast-containing medium developed towards a hgiher fitness in comparison to a blank medium or to one with gray rot. When the acetic acid bacteria were added to the medium, a similar fitness to the yeast-containing medium was measured.

While we have no information on the relation between attraction to single compound and larval fitness, it is intriguing that in the present study, gravid females were trapped with a binary blend of ubiquitous microbial compounds released either by all microbial combinations (2-phenylethanol) or by yeast and single or co-inoculated bacteria (acetic acid). Because this component was emitted with the highest amount by the repellent bacteria, it would be intuitive to exclude this compound from the candidate volatiles for field attraction. In fact, its attraction in the field as single components was not different from the blank. Similarly, 2-phenylethanol was inactive when presented alone. Although released at a very little amount in comparison with the major compounds, 2-phenylethanol may play a major behavioral role, as reported for other minor components [33].

While the emission of acetic acid from the yeasts was totally inhibited by the fungus in their co-inoculation, 3-methyl-1-butanol emerged as the second most abundant volatile after ethanol. According to these data, we may expect a stimulating effect of 3-methyl-1-butanol when co-occurring at a higher dose with other compounds such as ethyl acetate. The attractive properties of this alcohol are known for a number of insects [29, 34, 35]. When in the present study 3-methyl-1-butanol was presented in the field in combination with ethanol and ethyl acetate, no significant attraction was scored. However, although not significant, the ternary blend could have an additive effect on female captures when added on the top of 2-phenylethanol and acetic acid (Fig. 4). In the study of Tasin et al. (2012), the response of the grapevine moth to grapes with *B. cinerea* shifted from attraction to repulsion according to the time from inoculation. In the same study, 3-methyl-1-butanol was found to be repellent at a high dose while attractive at a low dose. We observed here that *L. botrana* females were not repelled when a blend of 30 mg of 3-methyl-1-butanol, ethyl acetate, and ethanol was added to the attractive binary mixture of acetic acid and 2-phenylethanol. From our result, the role of 3-methyl-1-butanol seems to be context-dependent on the presence of other constituents. The detrimental effect observed in Tasin et al. (2012) could have been reversed into an attractive stimulus by the addition of other volatiles. The new blend may represent to the insect a yeast related odor, which, according to the literature, should provide a higher fitness food to the offspring. The generalist feeding habit of *L. botrana* with populations interplaying between cultivated grape and other wild or cultivated plants adds further complexity to the observed yeast/fungus preference on grape.

Perhaps different volatiles are involved in triggering different behavioral functions, but the synergy between them is fundamental to elicit field attraction from a distance. While 2-phenylethanol could be relevant for both attraction and oviposition, acetic acid may elicit a rather longer-range attraction, because of its higher emission and potential to travel further from the source. While in the headspace from the inoculated berries the ratio between acetic acid and 2-phenylethanol ranged from 0.7 (yeasts) to 67 (bacteria), an intermediate ratio of 16 (load of the field lure in this study) was attractive in the field experiment. Although promising, our data form a preliminary base towards the identification of multicomponent field attractants, because a large number of minor compounds identified in the microbial headspace remain to be tested.

Recently, both acetic acid and 2-phenylethanol were scored in the headspace of damaged plants by different tortricid species as caterpillar induced volatiles [25]. These compounds were field attractive to conspecific adults across a range of moths, including *Pandemis* spp. and other tortricids [26]. It is intriguing that acetic acid and 2-phenylethanol were identified as behaviorally active both as microbial and caterpillar-induced plant volatiles. We speculate here that such a behavioral activity on a broad range of species may reflect a conserved behavioral pattern in Tortricidae, as shown for other olfactory traits in moths [36, 37]. According to the preference-performance hypothesis, it is predicted that herbivorous insects will evolve to lay eggs on hosts that will elicit the best performance in the offspring [38, 39]. Perhaps both microbial and caterpillar-induced volatiles are perceived by a searching insect as oviposition cues carrying an ecologically shared message, i.e., a nutritious substrate for the offspring.

Although plant volatiles were released in the oviposition arena, our laboratory experiment may be biased by the lower background of grapevine volatiles in comparison with a field situation. Accordingly, the preference observed in the laboratory may be shaped in a different way when the same experiment would be moved in a vineyard. The effect of grapevine volatiles on attraction and oviposition was earlier examined by Anfora et al. [40] in a semi-field setting through a release and recapture assay with gravid females. While green grapes were removed from the plants to eliminate the competition between the trapping odors and the fruits, only a small proportion of the released females were recaptured, with higher numbers in a synthetic grape mimic compared to a grape cluster [40].

In the same study, the synthetic mimic stimulated a higher oviposition on shoots surrounding the traps in comparison with the grape cluster. Overall, synthetic volatiles identified from the cultivated *V. vinifera* were not highly attractive to *L. botrana* females, probably due to a high degree of similarity with the background odor of the vineyard. *L. botrana* female may instead be attracted by an odor with a lower degree of similarity to grapevine, such as that released by other host plant or by microorganisms. While *L. botrana* wind tunnel response to artificial plant volatile mixtures with a higher attraction to *Daphne gnidium* compared to *V. vinifera* was examined, it is currently unknown whether or not such laboratory active compounds may play a role in a field setting [41]. Recently, a grapevine genotype with a distorted ratio of two terpenoids was created to show the effect of plant volatile ratio on grapevine moth attraction [42]. Such a result highlighted the importance of considering the ratio between volatiles when testing multicomponent blends in the field.

The potential role of microbial volatiles in overtaking the volatile background of the crop was demonstrated earlier in *L. botrana*. Field attraction of grapevine moth to fermenting apple juice was reported by Thiery and co-workers as a valuable tool to predict oviposition [43]. However, the fermentation of the initial product induced by air-borne microorganisms may lead to a large and unpredictable variation in the emission of volatiles over time. In addition, the attraction to water, which cannot be distinguished from the effect of volatiles, adds further variation to the efficacy of such a lure. Accordingly, the optimization of food lures through the identification of their volatile components seems to be a prerequisite to improve the reliability of such monitoring tool.

This study paved the way for the identification of field attracting volatiles for male and female grapevine moth. We showed here that a combination of major and minor volatile constituents is essential to reach this goal. In particular, a blend of a compound commonly released during microbial fermentation (acetic acid) with a volatile emitted by a number of flowering plant as well as by microbial activity (2-phenylethanol) encoded field attraction for the studied pest. The practical need to identify bisexual food attractants in this species was highlighted during its recent invasion of America along with its range expansion to new host species [44]. The identification of a kairomone for field monitoring is a relevant tool to facilitate the implementation of insecticide-free method and move towards an advanced integrated pest management of vineyards.

## Electronic supplementary material

Table S1 Volatile compounds (as percent of total ion abundance) detected in the headspace of grapes inoculated with different categories of microorganisms analyzed by SPME-GC-MS. (PDF 55 kb)
