# Supplementary material for: Volatiles of Grape Inoculated with Microorganisms: Modulation of Grapevine Moth Oviposition and Field Attraction
Source: Microb Ecol. 2018 Mar 10;76(3):751–61. doi: 10.1007/s00248-018-1164-6 (PMC6132500; doi:10.1007/s00248-018-1164-6)
Supplement: Supplementary file 1 — Volatile compounds (as percent of total ion abundance) detected in the headspace of grapes inoculated with different categories of microorganisms analyzed by SPME-GC-MS. (PDF 55 kb) [file 248_2018_1164_MOESM1_ESM.pdf]

Compound[b]	KI[c]	UG[d]	Treatments[a]						
			FYB	FB	FY	Y	F	YB	B
		Relative area in (%)							
Acetaldehyde	792	9.13	0.20	0.17	0.25	0.63	0.08	0.27	0.50
Acetone	815	54.07	0.02	0.50	0.04	0.41	0.08		0.08
Methyl Acetate	819		0.26		0.37	1.03		1.31	
Ethyl Acetate	842	6.04	12.96	0.29	19.04	54.36	0.53	68.72	0.91
Ethanol	877	7.18	46.43	63.70	42.61	26.89	86.84	9.66	44.68
Isobutyl acetate	940		0.19		0.24	0.30		0.59	
Isobutanol	1036		3.36	1.00	2.75	1.03	0.84	0.45	0.83
Isoamyl acetate	1053		0.59		0.73	1.06		2.87	
1-Butanol	1099		0.11		0.11	0.10	0.24		
Limonene	1137	8.16	0.76	1.56	0.69	0.58	1.45	0.32	1.41
Isoamyl Alcohol	1170		32.88	6.44	28.92	11.25	6.84	4.71	4.23
3-Methyl-3-buten-1-ol	1221		0.26	0.30	0.22	0.35	0.49	0.10	0.37
3-Octanone	1228		0.10	0.04	0.13	0.05	0.18	0.04	0.14
3-Hydroxy-2-butanone	1266		0.09	0.14	0.04	0.20		0.41	0.99
Hexanol	1349	9.81	0.38	0.79	0.57	0.36	0.18	0.11	0.91
2-Butoxy-ethanol	1411		0.33	0.45	2.26	0.16	0.60	0.07	0.26
1-Octen-3-ol	1466	0.88	0.21	0.51	0.44	0.57	0.95	0.30	1.29
Acetic Acid	1510			22.85		0.17		9.73	42.85
2-Ethyl-1-Hexanol	1512		0.21	0.16	0.09	0.17	0.27	0.02	
Benzaldehyde	1552		0.09	0.28	0.04	0.03	0.10	0.03	0.12
Butyrolactone	1668	2.90	0.14	0.42	0.05	0.04	0.15	0.05	0.19
Acetophenone	1693	0.73	0.04	0.08	0.02	0.01	0.04	0.01	0.04
Isovaleric Acid	1734							0.07	0.02
Methyl Salicilate	1825	0.36	0.03	0.06			0.01		0.02
Hexanoic Acid	1921		0.06	0.17	0.01	0.01	0.02	0.01	0.04
Benzilic Alcohol	1924	0.59	0.02	0.07	0.01	0.01	0.01	0.01	0.05
Phenylethyl Alcohol	1958	0.16	0.29	0.03	0.38	0.21	0.11	0.12	0.08

[a] B. cinerea (F), S. cerevisiae + Z. rouxii + M. pulcherrima + K. apiculata + H. anomala (Y), A. aceti + G. oxydans (B), FY (coinoculum of F+Y), FB (coinoculum of A+F), YB (coinoculum of Y+A), FYB (coinoculum of F+Y+A).

[b] Compound identified by correlation with mass spectra (Wiley library) and Kovats index.

[c] Kovats index on a Innowax (30m x 0.32mm x 0.5 Îijm) fused silica column.

[d] Uninoculated Grape, control
